# Supplementary material for: Rapid, Reference-Free human genotype imputation with denoising autoencoders
Source: eLife. 2022 Sep 23;11:e75600. doi: 10.7554/eLife.75600 (PMC9555874; doi:10.7554/eLife.75600)
Supplement: Supplementary file 1. [file elife-75600-supp1.docx]

**Supplementary File 1.** Performance comparisons between tuned autoencoder (AE) and HMM-based imputation tools (Minimac4, Beagle5, and Impute5) after applying data augmentation to HMM-based tools.

|  | **MESA** | **Wellderly** | **HGDP** | **Affymetrix 6.0** | **UKB Axiom** | **Omni 1.5M** | **Combined** |
| --- | --- | --- | --- | --- | --- | --- | --- |
| **AE (tuned) vs Minimac4 (augmented)** | 1.36e-04* | 3.49e-06* | 1.18e-03* | 6.05e-04* | 6.98e-08* | 1.95e-03* | 3.39e-05* |
| **AE (tuned) vs Beagle5 (augmented)** | 1.71e-05* | 1.68e-09* | 2.88e-09* | 1.54e-06* | 3.94e-10* | 4.30e-07* | 2.30e-08* |
| **AE (tuned) vs Impute5 (augmented)** | 1.24e-09* | 3.15e-15* | 5.28e-15* | 4.41e-11* | 2.47e-18* | 4.90e-10* | 8.64e-14* |
| **Minimac4 (original vs augmented)** | 4.91e-02* | 2.07E-01 | 1.03E-01 | 1.74E-01 | 4.36e-02* | 1.13E-01 | 9.17E-02 |
| **Beagle5 (original vs augmented)** | 1.21e-02* | 8.21E-02 | 2.35e-02* | 8.96E-02 | 6.59e-03* | 5.27E-02 | 2.58e-02* |
| **Impute5 (original vs augmented)** | 5.45e-04* | 6.89e-05* | 1.78e-04* | 7.01e-04* | 1.16e-05* | 4.15e-04* | 1.26e-04* |
| **AE (tuned)** | 0.355±0.007 | 0.505±0.008 | 0.327±0.006 | 0.373±0.008 | 0.399±0.007 | 0.414±0.008 | 0.396±0.007 |
| **Minimac4 (augmented)** | 0.322±0.007 | 0.462±0.008 | 0.303±0.006 | 0.342±0.008 | 0.358±0.006 | 0.388±0.007 | 0.363±0.007 |
| **Beagle5 (augmented)** | 0.316±0.007 | 0.446±0.008 | 0.283±0.005 | 0.327±0.007 | 0.348±0.006 | 0.370±0.007 | 0.349±0.006 |
| **Impute5 (augmented)** | 0.294±0.007 | 0.416±0.008 | 0.261±0.006 | 0.302±0.008 | 0.318±0.006 | 0.351±0.008 | 0.324±0.007 |

We applied Wilcoxon rank-sum tests to compare the HMM-based tools to the reference tuned autoencoder (AE). * represents p-values ≤ 0.05, ** indicates p-values ≤ 0.001, and *** indicates p-values ≤ 0.0001
