# Supplementary material for: Rapid, Reference-Free human genotype imputation with denoising autoencoders
Source: eLife. 2022 Sep 23;11:e75600. doi: 10.7554/eLife.75600 (PMC9555874; doi:10.7554/eLife.75600)
Supplement: Supplementary file 2. [file elife-75600-supp2.docx]

**Supplementary File 2.** Detailed performance comparisons between tuned autoencoder (AE) and HMM-based imputation tools (Minimac4, Beagle5, and Impute5).

| **Dataset** | **MESA** | | | **Wellderly** | | | **HGDP** | | |
| --- | --- | --- | --- | --- | --- | --- | --- | --- | --- |
| **array** | **Affymetrix 6.0** | **UKB Axiom** | **Omni 1.5M** | **Affymetrix 6.0** | **UKB Axiom** | **Omni 1.5M** | **Affymetrix 6.0** | **UKB Axiom** | **Omni 1.5M** |
| **AE (tuned) vs Minimac4** | 9.47e-185*** | 0.00e+00*** | 7.22e-89*** | 6.27e-209*** | 0.00e+00*** | 2.75e-198*** | 2.35e-151*** | 0.00e+00*** | 5.55e-67*** |
| **AE (tuned) vs Beagle5** | 0.00e+00*** | 0.00e+00*** | 0.00e+00*** | 0.00e+00*** | 0.00e+00*** | 0.00e+00*** | 0.00e+00*** | 0.00e+00*** | 0.00e+00*** |
| **AE (tuned) vs Impute5** | 0.00e+00*** | 0.00e+00*** | 5.37e-191*** | 0.00e+00*** | 0.00e+00*** | 0.00e+00*** | 0.00e+00*** | 0.00e+00*** | 0.00e+00*** |
| **Minimac4 vs Beagle5** | 1.73e-259*** | 6.06e-16*** | 0.00e+00*** | 2.68e-86*** | 1.65e-11*** | 6.87e-64*** | 0.00e+00*** | 2.62e-185*** | 0.00e+00*** |
| **Minimac4 vs Impute5** | 4.87e-38*** | 3.59e-48*** | 1.17e-22*** | 3.05e-74*** | 5.94e-15*** | 1.00e-25*** | 9.43e-261*** | 0.00e+00*** | 1.73e-251*** |
| **Beagle5 vs Impute5** | 1.92e-96*** | 2.75e-09*** | 1.23e-175*** | 3.65E-01 | 1.98E-01 | 9.53e-09*** | 1.22e-25*** | 2.61e-17*** | 8.36e-57*** |
| **AE (tuned)** | 0.410±0.001 | 0.395±0.001 | 0.452±0.001 | 0.537±0.001 | 0.605±0.001 | 0.586±0.001 | 0.363±0.001 | 0.364±0.001 | 0.392±0.001 |
| **Minimac4** | 0.390±0.001 | 0.364±0.001 | 0.436±0.001 | 0.500±0.001 | 0.557±0.001 | 0.551±0.001 | 0.350±0.001 | 0.340±0.001 | 0.385±0.001 |
| **Beagle5** | 0.383±0.001 | 0.379±0.001 | 0.420±0.001 | 0.484±0.001 | 0.549±0.001 | 0.534±0.001 | 0.326±0.001 | 0.328±0.001 | 0.353±0.001 |
| **Impute5** | 0.384±0.001 | 0.356±0.001 | 0.429±0.001 | 0.485±0.001 | 0.547±0.001 | 0.539±0.001 | 0.328±0.001 | 0.314±0.001 | 0.359±0.001 |

Validation accuracies were stratified by dataset (MESA, Wellderly, HGDP) and genotype array platform (Affymetrix 6.0, UKB Axiom, Omni 1.5M). We applied Wilcoxon rank-sum tests to compare the HMM-based tools to the reference tuned autoencoder (AE). * represents p-values ≤ 0.05, ** indicates p-values ≤ 0.001, and *** indicates p-values ≤ 0.0001
