# Supplementary material for: Rapid, Reference-Free human genotype imputation with denoising autoencoders
Source: eLife. 2022 Sep 23;11:e75600. doi: 10.7554/eLife.75600 (PMC9555874; doi:10.7554/eLife.75600)
Supplement: Supplementary file 3. [file elife-75600-supp3.docx]

**Supplementary File 3.** Detailed performance comparisons between tuned autoencoder (AE) and HMM-based imputation tools (Minimac4, Beagle5, and Impute5).

| **Dataset** | **Array** | **MAF** | **AE (tuned) vs Minimac4** | **AE (tuned) vs Beagle5** | **AE (tuned) vs Impute5** | **Minimac4 vs Beagle5** | **Minimac4 vs Impute5** | **Beagle5 vs Impute5** | **AE (tuned)** | **Minimac4** | **Beagle5** | **Impute5** |
| --- | --- | --- | --- | --- | --- | --- | --- | --- | --- | --- | --- | --- |
| **MESA** | **Affymetrix 6.0** | **[0.001-0.005)** | 3.84e-306*** | 0.00e+00*** | 0.00e+00*** | 0.00e+00*** | 1.30e-122*** | 0.00e+00*** | 0.141±0.001 | 0.128±0.001 | 0.129±0.001 | 0.121±0.001 |
| **MESA** | **Affymetrix 6.0** | **[0.005-0.01)** | 1.37e-48*** | 3.39e-280*** | 8.96e-86*** | 3.12e-127*** | 1.13e-08*** | 1.22e-68*** | 0.280±0.001 | 0.266±0.001 | 0.258±0.001 | 0.262±0.001 |
| **MESA** | **Affymetrix 6.0** | **[0.01-0.05)** | 2.62e-48*** | 6.14e-119*** | 3.54e-72*** | 4.61e-23*** | 8.85e-05*** | 7.81e-09*** | 0.490±0.001 | 0.467±0.001 | 0.453±0.001 | 0.461±0.001 |
| **MESA** | **Affymetrix 6.0** | **[0.05-0.1)** | 2.11e-05*** | 4.44e-49*** | 2.36e-04** | 3.26e-23*** | 7.11E-01 | 3.53e-24*** | 0.728±0.002 | 0.703±0.002 | 0.684±0.002 | 0.698±0.002 |
| **MESA** | **Affymetrix 6.0** | **[0.1-0.2)** | 1.17e-15*** | 2.04e-50*** | 3.36e-09*** | 6.41e-11*** | 6.50E-02 | 8.64e-16*** | 0.793±0.002 | 0.763±0.002 | 0.753±0.002 | 0.758±0.002 |
| **MESA** | **Affymetrix 6.0** | **[0.2-0.3)** | 1.02e-16*** | 5.61e-25*** | 6.87e-09*** | 5.09E-02 | 2.67e-02* | 5.57e-05*** | 0.825±0.002 | 0.794±0.002 | 0.790±0.002 | 0.789±0.002 |
| **MESA** | **Affymetrix 6.0** | **[0.3-0.4)** | 2.41e-19*** | 3.04e-28*** | 2.92e-09*** | 9.05E-02 | 1.08e-02* | 3.17e-05*** | 0.834±0.002 | 0.799±0.002 | 0.798±0.002 | 0.795±0.002 |
| **MESA** | **Affymetrix 6.0** | **[0.4-0.5)** | 2.46e-18*** | 7.67e-23*** | 3.85e-11*** | 3.80E-01 | 7.17E-02 | 9.77e-03* | 0.842±0.002 | 0.806±0.002 | 0.805±0.003 | 0.801±0.003 |
| **MESA** | **UKB Axiom** | **[0.001-0.005)** | 0.00e+00*** | 0.00e+00*** | 0.00e+00*** | 0.00e+00*** | 1.04e-106*** | 1.10e-95*** | 0.145±0.001 | 0.117±0.001 | 0.132±0.001 | 0.108±0.001 |
| **MESA** | **UKB Axiom** | **[0.005-0.01)** | 7.45e-142*** | 7.16e-191*** | 4.41e-242*** | 2.76e-23*** | 8.03e-18*** | 1.28E-01 | 0.255±0.001 | 0.226±0.001 | 0.249±0.001 | 0.216±0.001 |
| **MESA** | **UKB Axiom** | **[0.01-0.05)** | 2.85e-128*** | 6.38e-41*** | 1.57e-181*** | 1.76e-12*** | 3.39e-07*** | 2.75e-34*** | 0.432±0.001 | 0.400±0.001 | 0.418±0.001 | 0.393±0.001 |
| **MESA** | **UKB Axiom** | **[0.05-0.1)** | 5.91e-21*** | 4.88e-09*** | 2.14e-23*** | 2.68e-03* | 4.80E-01 | 3.13e-04** | 0.681±0.002 | 0.652±0.002 | 0.657±0.002 | 0.646±0.002 |
| **MESA** | **UKB Axiom** | **[0.1-0.2)** | 1.12e-42*** | 2.66e-11*** | 5.58e-37*** | 9.55e-11*** | 5.04E-01 | 2.58e-08*** | 0.791±0.001 | 0.758±0.002 | 0.766±0.002 | 0.752±0.002 |
| **MESA** | **UKB Axiom** | **[0.2-0.3)** | 8.25e-59*** | 1.62e-15*** | 5.06e-54*** | 2.23e-14*** | 8.15E-01 | 1.07e-12*** | 0.837±0.001 | 0.796±0.002 | 0.807±0.002 | 0.790±0.002 |
| **MESA** | **UKB Axiom** | **[0.3-0.4)** | 8.13e-81*** | 7.00e-14*** | 3.82e-72*** | 3.98e-26*** | 8.34E-01 | 2.26e-22*** | 0.840±0.002 | 0.795±0.002 | 0.810±0.002 | 0.789±0.002 |
| **MESA** | **UKB Axiom** | **[0.4-0.5)** | 5.60e-82*** | 8.03e-12*** | 1.69e-79*** | 6.34e-30*** | 6.20E-01 | 3.13e-29*** | 0.846±0.002 | 0.800±0.002 | 0.819±0.002 | 0.794±0.002 |
| **MESA** | **Omni 1.5M** | **[0.001-0.005)** | 6.78e-179*** | 0.00e+00*** | 0.00e+00*** | 0.00e+00*** | 8.51e-66*** | 0.00e+00*** | 0.174±0.001 | 0.158±0.001 | 0.152±0.001 | 0.148±0.001 |
| **MESA** | **Omni 1.5M** | **[0.005-0.01)** | 1.37e-23*** | 7.31e-253*** | 5.27e-53*** | 6.68e-147*** | 5.95e-08*** | 1.44e-90*** | 0.340±0.001 | 0.327±0.001 | 0.301±0.002 | 0.317±0.001 |
| **MESA** | **Omni 1.5M** | **[0.01-0.05)** | 1.51e-05*** | 6.15e-118*** | 9.61e-14*** | 2.01e-77*** | 1.54e-03* | 6.53e-53*** | 0.552±0.001 | 0.542±0.001 | 0.510±0.001 | 0.537±0.001 |
| **MESA** | **Omni 1.5M** | **[0.05-0.1)** | 8.96E-01 | 2.53e-52*** | 1.91E-01 | 2.25e-48*** | 2.57E-01 | 2.05e-54*** | 0.759±0.002 | 0.750±0.002 | 0.723±0.002 | 0.749±0.002 |
| **MESA** | **Omni 1.5M** | **[0.1-0.2)** | 7.72e-19*** | 3.14e-57*** | 5.03e-09*** | 5.40e-11*** | 6.88e-03* | 2.65e-19*** | 0.828±0.001 | 0.806±0.002 | 0.797±0.002 | 0.805±0.002 |
| **MESA** | **Omni 1.5M** | **[0.2-0.3)** | 2.53e-32*** | 2.68e-65*** | 6.69e-18*** | 8.66e-06*** | 6.61e-03* | 6.35e-12*** | 0.866±0.002 | 0.838±0.002 | 0.834±0.002 | 0.838±0.002 |
| **MESA** | **Omni 1.5M** | **[0.3-0.4)** | 4.87e-32*** | 3.52e-34*** | 5.66e-20*** | 8.88E-01 | 3.34e-02* | 2.46e-02* | 0.864±0.002 | 0.836±0.002 | 0.835±0.002 | 0.834±0.002 |
| **MESA** | **Omni 1.5M** | **[0.4-0.5)** | 2.05e-53*** | 1.11e-22*** | 1.92e-38*** | 3.17e-08*** | 7.47E-02 | 4.31e-04** | 0.879±0.002 | 0.847±0.002 | 0.854±0.002 | 0.845±0.002 |
| **Wellderly** | **Affymetrix 6.0** | **[0.001-0.005)** | 0.00e+00*** | 0.00e+00*** | 0.00e+00*** | 0.00e+00*** | 0.00e+00*** | 5.06e-19*** | 0.212±0.001 | 0.183±0.001 | 0.170±0.001 | 0.170±0.001 |
| **Wellderly** | **Affymetrix 6.0** | **[0.005-0.01)** | 9.46e-58*** | 4.82e-140*** | 2.37e-167*** | 1.84e-26*** | 1.44e-39*** | 1.40e-02* | 0.359±0.003 | 0.307±0.003 | 0.289±0.003 | 0.283±0.003 |
| **Wellderly** | **Affymetrix 6.0** | **[0.01-0.05)** | 4.01e-43*** | 2.50e-149*** | 1.02e-88*** | 6.52e-34*** | 9.91e-12*** | 1.85e-06*** | 0.616±0.002 | 0.566±0.002 | 0.536±0.002 | 0.544±0.002 |
| **Wellderly** | **Affymetrix 6.0** | **[0.05-0.1)** | 4.80e-19*** | 2.25e-55*** | 7.24e-16*** | 7.05e-12*** | 5.76E-01 | 7.95e-12*** | 0.820±0.002 | 0.783±0.003 | 0.761±0.003 | 0.769±0.003 |
| **Wellderly** | **Affymetrix 6.0** | **[0.1-0.2)** | 1.08e-46*** | 4.14e-53*** | 3.92e-34*** | 2.40E-01 | 1.23E-01 | 1.03e-02* | 0.869±0.002 | 0.830±0.002 | 0.821±0.002 | 0.820±0.002 |
| **Wellderly** | **Affymetrix 6.0** | **[0.2-0.3)** | 7.27e-35*** | 4.70e-38*** | 1.12e-23*** | 5.90E-01 | 9.11E-02 | 2.47e-02* | 0.889±0.002 | 0.856±0.002 | 0.850±0.002 | 0.848±0.002 |
| **Wellderly** | **Affymetrix 6.0** | **[0.3-0.4)** | 1.93e-49*** | 3.99e-32*** | 1.43e-35*** | 7.35e-03* | 1.17E-01 | 3.62E-01 | 0.888±0.002 | 0.851±0.002 | 0.848±0.003 | 0.844±0.002 |
| **Wellderly** | **Affymetrix 6.0** | **[0.4-0.5)** | 8.77e-51*** | 7.23e-32*** | 5.15e-31*** | 3.34e-03* | 1.11e-02* | 8.04E-01 | 0.900±0.002 | 0.861±0.002 | 0.859±0.002 | 0.855±0.002 |
| **Wellderly** | **UKB Axiom** | **[0.001-0.005)** | 0.00e+00*** | 0.00e+00*** | 0.00e+00*** | 1.56e-98*** | 2.93e-173*** | 5.08e-13*** | 0.240±0.001 | 0.188±0.001 | 0.179±0.001 | 0.176±0.001 |
| **Wellderly** | **UKB Axiom** | **[0.005-0.01)** | 1.42e-78*** | 3.64e-137*** | 4.44e-134*** | 8.25e-14*** | 2.51e-11*** | 3.79E-01 | 0.463±0.003 | 0.392±0.003 | 0.374±0.003 | 0.373±0.003 |
| **Wellderly** | **UKB Axiom** | **[0.01-0.05)** | 3.20e-43*** | 4.20e-124*** | 1.06e-54*** | 5.93e-22*** | 3.75e-02* | 1.29e-12*** | 0.761±0.002 | 0.714±0.002 | 0.694±0.002 | 0.701±0.002 |
| **Wellderly** | **UKB Axiom** | **[0.05-0.1)** | 1.63e-62*** | 1.70e-80*** | 1.47e-32*** | 6.88e-03* | 7.27e-05*** | 1.74e-10*** | 0.911±0.001 | 0.879±0.002 | 0.868±0.002 | 0.875±0.002 |
| **Wellderly** | **UKB Axiom** | **[0.1-0.2)** | 1.21e-141*** | 4.36e-96*** | 4.21e-87*** | 1.88e-04** | 2.59e-05*** | 5.92E-01 | 0.930±0.001 | 0.893±0.001 | 0.891±0.001 | 0.889±0.001 |
| **Wellderly** | **UKB Axiom** | **[0.2-0.3)** | 4.84e-219*** | 7.82e-121*** | 4.78e-154*** | 9.07e-15*** | 9.63e-04** | 3.48e-05*** | 0.944±0.001 | 0.902±0.001 | 0.907±0.001 | 0.899±0.001 |
| **Wellderly** | **UKB Axiom** | **[0.3-0.4)** | 5.32e-257*** | 3.33e-135*** | 1.46e-196*** | 1.38e-21*** | 4.46e-02* | 4.42e-12*** | 0.940±0.001 | 0.892±0.002 | 0.899±0.002 | 0.886±0.002 |
| **Wellderly** | **UKB Axiom** | **[0.4-0.5)** | 0.00e+00*** | 5.32e-156*** | 1.60e-244*** | 1.62e-34*** | 1.02e-02* | 6.59e-19*** | 0.949±0.001 | 0.900±0.001 | 0.908±0.002 | 0.895±0.002 |
| **Wellderly** | **Omni 1.5M** | **[0.001-0.005)** | 0.00e+00*** | 0.00e+00*** | 0.00e+00*** | 2.69e-183*** | 1.06e-207*** | 7.98e-03* | 0.255±0.001 | 0.215±0.001 | 0.197±0.001 | 0.203±0.001 |
| **Wellderly** | **Omni 1.5M** | **[0.005-0.01)** | 1.20e-46*** | 1.49e-125*** | 1.09e-102*** | 8.69e-25*** | 4.07e-15*** | 1.55e-02* | 0.428±0.003 | 0.376±0.003 | 0.347±0.003 | 0.355±0.003 |
| **Wellderly** | **Omni 1.5M** | **[0.01-0.05)** | 1.67e-13*** | 1.07e-93*** | 1.95e-25*** | 2.72e-36*** | 1.68e-03* | 7.71e-19*** | 0.706±0.002 | 0.671±0.002 | 0.641±0.002 | 0.653±0.002 |
| **Wellderly** | **Omni 1.5M** | **[0.05-0.1)** | 5.40e-07*** | 6.60e-59*** | 8.37e-04** | 1.59e-26*** | 1.12E-01 | 1.08e-31*** | 0.883±0.002 | 0.862±0.002 | 0.844±0.002 | 0.854±0.002 |
| **Wellderly** | **Omni 1.5M** | **[0.1-0.2)** | 2.89e-42*** | 1.73e-55*** | 1.08e-22*** | 4.85e-02* | 1.70e-03* | 1.76e-06*** | 0.915±0.002 | 0.889±0.002 | 0.885±0.002 | 0.883±0.002 |
| **Wellderly** | **Omni 1.5M** | **[0.2-0.3)** | 2.56e-68*** | 6.21e-68*** | 9.93e-47*** | 8.71E-01 | 3.06e-02* | 6.20E-02 | 0.933±0.001 | 0.907±0.002 | 0.904±0.002 | 0.901±0.002 |
| **Wellderly** | **Omni 1.5M** | **[0.3-0.4)** | 3.99e-89*** | 1.54e-58*** | 3.55e-68*** | 1.15e-04** | 1.52E-01 | 3.57e-02* | 0.927±0.002 | 0.896±0.002 | 0.897±0.002 | 0.892±0.002 |
| **Wellderly** | **Omni 1.5M** | **[0.4-0.5)** | 1.88e-101*** | 5.27e-60*** | 2.12e-68*** | 6.34e-07*** | 1.44e-02* | 2.86e-02* | 0.933±0.002 | 0.902±0.002 | 0.904±0.002 | 0.897±0.002 |
| **HGDP** | **Affymetrix 6.0** | **[0.001-0.005)** | 0.00e+00*** | 0.00e+00*** | 0.00e+00*** | 0.00e+00*** | 0.00e+00*** | 4.57e-76*** | 0.115±0.000 | 0.110±0.001 | 0.094±0.000 | 0.097±0.000 |
| **HGDP** | **Affymetrix 6.0** | **[0.005-0.01)** | 9.42e-37*** | 0.00e+00*** | 1.41e-180*** | 1.23e-172*** | 2.29e-59*** | 6.90e-31*** | 0.255±0.001 | 0.245±0.001 | 0.212±0.001 | 0.224±0.001 |
| **HGDP** | **Affymetrix 6.0** | **[0.01-0.05)** | 1.27e-19*** | 5.35e-251*** | 5.51e-161*** | 3.40e-124*** | 1.46e-68*** | 3.88e-09*** | 0.477±0.001 | 0.461±0.001 | 0.416±0.001 | 0.428±0.001 |
| **HGDP** | **Affymetrix 6.0** | **[0.05-0.1)** | 5.84e-07*** | 1.05e-90*** | 3.49e-67*** | 5.86e-46*** | 3.24e-32*** | 4.07e-02* | 0.720±0.002 | 0.695±0.002 | 0.662±0.002 | 0.660±0.002 |
| **HGDP** | **Affymetrix 6.0** | **[0.1-0.2)** | 4.01e-09*** | 6.45e-78*** | 6.97e-76*** | 1.44e-32*** | 1.49e-33*** | 4.55E-01 | 0.791±0.002 | 0.765±0.002 | 0.747±0.002 | 0.733±0.002 |
| **HGDP** | **Affymetrix 6.0** | **[0.2-0.3)** | 2.52e-13*** | 1.54e-56*** | 2.43e-64*** | 9.52e-15*** | 2.08e-20*** | 6.08E-02 | 0.813±0.002 | 0.786±0.002 | 0.774±0.002 | 0.760±0.002 |
| **HGDP** | **Affymetrix 6.0** | **[0.3-0.4)** | 5.85e-16*** | 6.47e-64*** | 7.68e-72*** | 2.25e-15*** | 9.49e-21*** | 7.35E-02 | 0.835±0.002 | 0.806±0.002 | 0.793±0.002 | 0.782±0.002 |
| **HGDP** | **Affymetrix 6.0** | **[0.4-0.5)** | 2.89e-16*** | 7.59e-40*** | 9.69e-63*** | 7.01e-06*** | 3.98e-16*** | 7.45e-05*** | 0.836±0.002 | 0.805±0.002 | 0.797±0.002 | 0.781±0.002 |
| **HGDP** | **UKB Axiom** | **[0.001-0.005)** | 0.00e+00*** | 0.00e+00*** | 0.00e+00*** | 0.00e+00*** | 0.00e+00*** | 1.59e-03* | 0.109±0.000 | 0.096±0.000 | 0.086±0.000 | 0.080±0.000 |
| **HGDP** | **UKB Axiom** | **[0.005-0.01)** | 6.42e-154*** | 0.00e+00*** | 0.00e+00*** | 1.60e-75*** | 4.56e-76*** | 5.59E-01 | 0.233±0.001 | 0.206±0.001 | 0.193±0.001 | 0.180±0.001 |
| **HGDP** | **UKB Axiom** | **[0.01-0.05)** | 1.58e-100*** | 7.46e-277*** | 0.00e+00*** | 7.30e-49*** | 2.07e-107*** | 1.88e-12*** | 0.455±0.001 | 0.421±0.001 | 0.396±0.001 | 0.382±0.001 |
| **HGDP** | **UKB Axiom** | **[0.05-0.1)** | 9.25e-18*** | 8.34e-48*** | 3.52e-139*** | 2.05e-08*** | 9.53e-57*** | 2.15e-24*** | 0.746±0.002 | 0.718±0.002 | 0.704±0.002 | 0.678±0.002 |
| **HGDP** | **UKB Axiom** | **[0.1-0.2)** | 1.69e-51*** | 1.48e-73*** | 1.93e-253*** | 3.65e-03* | 9.55e-77*** | 3.82e-55*** | 0.817±0.001 | 0.785±0.001 | 0.779±0.001 | 0.748±0.002 |
| **HGDP** | **UKB Axiom** | **[0.2-0.3)** | 2.31e-71*** | 5.99e-63*** | 4.54e-249*** | 3.50E-01 | 2.43e-55*** | 2.29e-60*** | 0.844±0.001 | 0.808±0.002 | 0.805±0.002 | 0.773±0.002 |
| **HGDP** | **UKB Axiom** | **[0.3-0.4)** | 1.63e-104*** | 4.84e-81*** | 3.67e-302*** | 9.67e-03* | 2.63e-52*** | 3.67e-70*** | 0.860±0.001 | 0.819±0.002 | 0.818±0.002 | 0.788±0.002 |
| **HGDP** | **UKB Axiom** | **[0.4-0.5)** | 3.74e-93*** | 5.24e-50*** | 8.92e-262*** | 5.86e-08*** | 2.88e-45*** | 3.80e-83*** | 0.861±0.001 | 0.821±0.002 | 0.827±0.002 | 0.791±0.002 |
| **HGDP** | **Omni 1.5M** | **[0.001-0.005)** | 5.46e-226*** | 0.00e+00*** | 0.00e+00*** | 0.00e+00*** | 3.78e-302*** | 1.17e-153*** | 0.139±0.001 | 0.135±0.001 | 0.112±0.001 | 0.117±0.001 |
| **HGDP** | **Omni 1.5M** | **[0.005-0.01)** | 1.04e-09*** | 0.00e+00*** | 2.39e-110*** | 2.55e-221*** | 2.71e-56*** | 1.73e-59*** | 0.292±0.001 | 0.290±0.001 | 0.240±0.001 | 0.261±0.001 |
| **HGDP** | **Omni 1.5M** | **[0.01-0.05)** | 4.61E-01 | 7.26e-252*** | 8.61e-99*** | 1.15e-221*** | 3.08e-85*** | 2.14e-34*** | 0.513±0.001 | 0.510±0.001 | 0.451±0.001 | 0.474±0.001 |
| **HGDP** | **Omni 1.5M** | **[0.05-0.1)** | 1.58E-01 | 5.99e-77*** | 2.18e-41*** | 5.10e-79*** | 2.87e-45*** | 2.85e-05*** | 0.772±0.002 | 0.764±0.002 | 0.730±0.002 | 0.730±0.002 |
| **HGDP** | **Omni 1.5M** | **[0.1-0.2)** | 1.63e-08*** | 3.95e-95*** | 7.52e-111*** | 1.44e-43*** | 6.73e-57*** | 5.16e-03* | 0.822±0.001 | 0.805±0.002 | 0.787±0.002 | 0.774±0.002 |
| **HGDP** | **Omni 1.5M** | **[0.2-0.3)** | 2.18e-14*** | 5.45e-80*** | 1.70e-107*** | 2.92e-25*** | 5.09e-43*** | 7.27e-05*** | 0.851±0.002 | 0.832±0.002 | 0.819±0.002 | 0.805±0.002 |
| **HGDP** | **Omni 1.5M** | **[0.3-0.4)** | 1.09e-34*** | 1.27e-114*** | 5.18e-157*** | 7.10e-21*** | 4.34e-44*** | 4.80e-07*** | 0.873±0.001 | 0.848±0.002 | 0.836±0.002 | 0.821±0.002 |
| **HGDP** | **Omni 1.5M** | **[0.4-0.5)** | 2.78e-29*** | 8.21e-50*** | 4.50e-131*** | 1.90e-03* | 2.67e-37*** | 1.11e-23*** | 0.867±0.002 | 0.843±0.002 | 0.837±0.002 | 0.817±0.002 |

Validation accuracies were stratified by dataset (MESA, Wellderly, HGDP), genotype array platform (Affymetrix 6.0, UKB Axiom, Omni 1.5M), and MAF bin. We applied Wilcoxon rank-sum tests to compare the HMM-based tools to the reference tuned autoencoder (AE). * represents p-values ≤ 0.05, ** indicates p-values ≤ 0.001, and *** indicates p-values ≤ 0.0001.
